# Controlling disease outbreaks in wildlife using limited culling: modelling classical swine fever incursions in wild pigs in Australia

**DOI:** 10.1186/1297-9716-43-3

**Published:** 2012-01-16

**Authors:** Brendan D Cowled, M Graeme Garner, Katherine Negus, Michael P Ward

**Affiliations:** 1 The Faculty of Veterinary Science, The University of Sydney, NSW, Australia, 2570; 2 The Australian Government Department of Agriculture, Fisheries and Forestry, GPO Box 858, Canberra, ACT, Australia, 2601

## Abstract

Disease modelling is one approach for providing new insights into wildlife disease epidemiology. This paper describes a spatio-temporal, stochastic, susceptible- exposed-infected-recovered process model that simulates the potential spread of classical swine fever through a documented, large and free living wild pig population following a simulated incursion. The study area (300 000 km^2^) was in northern Australia. Published data on wild pig ecology from Australia, and international Classical Swine Fever data was used to parameterise the model. Sensitivity analyses revealed that herd density (best estimate 1-3 pigs km^-2^), daily herd movement distances (best estimate approximately 1 km), probability of infection transmission between herds (best estimate 0.75) and disease related herd mortality (best estimate 42%) were highly influential on epidemic size but that extraordinary movements of pigs and the yearly home range size of a pig herd were not. CSF generally established (98% of simulations) following a single point introduction. CSF spread at approximately 9 km^2 ^per day with low incidence rates (< 2 herds per day) in an epidemic wave along contiguous habitat for several years, before dying out (when the epidemic arrived at the end of a contiguous sub-population or at a low density wild pig area). The low incidence rate indicates that surveillance for wildlife disease epidemics caused by short lived infections will be most efficient when surveillance is based on detection and investigation of clinical events, although this may not always be practical. Epidemics could be contained and eradicated with culling (aerial shooting) or vaccination when these were adequately implemented. It was apparent that the spatial structure, ecology and behaviour of wild populations must be accounted for during disease management in wildlife. An important finding was that it may only be necessary to cull or vaccinate relatively small proportions of a population to successfully contain and eradicate some wildlife disease epidemics.

## Introduction

Wildlife infectious disease can have enormous ecological, biodiversity and societal impacts [1-4]. However, management responses required for mitigation are frequently limited by poor understanding of wildlife disease epidemiology.

Disease modelling is one approach for providing new insights into wildlife disease epidemiology and has allowed important conceptual advances in wildlife disease management [5]. Mathematical modelling was an early method used (and is still widely applied) [6-9]. However, application of this method has often been simplistic, not incorporating many of the major ecological factors that affect disease epidemiology [10]. Furthermore, one of the key concepts in mathematical models - the existence of a threshold level of host abundance required for invasion or persistence of infection - originated in human health and is poorly supported by evidence from wildlife disease studies [11].

With the improvement of information technology, process models (or simulation models) have been advocated by some as a method of more realistically representing the complexity of real world animal health problems [12,13]. Process models can capture great complexity, thus enhancing our ability to model complex situations. These models have been widely applied in animal health generally, but relatively less commonly in wildlife disease epidemiology, with some exceptions [14-19].

To take advantage of the great complexity that process models can incorporate, a good understanding of the "process" (host-infection interaction) is required. *Sus scrofa*, commonly known as wild boar, feral pig, wild hog and wild pig (herein referred to as wild pig) is an important international wildlife species found on every continent except Antarctica [20]. Considerable research has been conducted internationally on wild pig ecology [21-25], and this research can be harnessed to construct detailed process models to study disease epidemiology in this species. Wild pigs have been involved in the transmission or maintenance of many agriculturally important infectious trans-boundary diseases such as African swine fever [26], pseudorabies [27] and foot-and-mouth disease [28], as well as the spread of important endemic zoonoses such as *Brucella suis *[29]. Classical swine fever (CSF) is another important trans-boundary agricultural disease of domestic and wild pigs [30]. Outbreaks of CSF in Europe have cost many billions of dollars to eradicate [31], and cause ongoing costs in areas where it is endemic. CSF also has a wide geographic distribution, being found in Asia, Europe, parts of Africa and central and South America [30], but not Australia. Wild pigs are frequently important in the epidemiology of CSF [32], but the issue is complex as demonstrated by Boklund et al. [33] who investigated the potential role of wild boar in CSF epidemics in Denmark.

Some limited epidemiological modelling of CSF in wild pigs has been conducted. Hone and Yip [34] estimated model parameters with field data and used a mathematical modelling approach to study CSF in wild pig populations. They found that CSF will establish in a small population of wild pigs. Milne et al. [35], using a process modelling approach found that seasonality is important in dispersal of CSF during epidemics but made some significant logical errors in formulating their model (for example, that wild pigs will be attracted to water only every 4-8 days in the extremely hot Australian sub-tropics). Kramer-Schadt et al. [36] conducted a review and used a conceptual model to putatively identify the reasons that CSF can persist in some populations. They found virulence of CSF and the size and structure of a wild pig population to be important. They used spatial modelling to show that individual level variation in infection persistence and production of new susceptible individuals was important for disease persistence [37]. Boklund et al. [33] found a complex epidemiology for CSF outbreaks where wild boar and domestic herds co-exist.

The objective of the research reported in this paper was to enhance knowledge of wildlife disease ecology and assess some control techniques for eradicating disease in wildlife. The paper first describes a spatio-temporal, stochastic, susceptible-exposed-infected-recovered process model that simulates the potential spread of classical swine fever through a well documented, large and free living wild pig population in Australia (which is free of CSF). Results are then used to explore disease ecology and control of CSF in free living wild pigs.

## Materials and methods

### Method summary

This study focused on a large wild pig population in a remote area of north-west Australia. It simulated the introduction of a virulent CSF virus into the population to explore epidemic behaviour, disease ecology and various epidemic control options. Three simulation models were developed. Model 1 was a non-spatial within-herd model in which the unit of interest was individual wild pigs (this model is summarised in Additional file 1; results are presented in Table 1). This model was simply used to estimate herd-based epidemiological parameters (i.e. convert individual parameters such as individual infectious period to a herd based parameter). These herd based parameters were then used in a between-herd model (Model 2) which was the main focus of the paper. Model 2 simulated the spread of CSF across a population of wild pig herds in time and space. In Model 2, herds ranged from individual boars to a group of co-mingling wild pigs occupying a territory or home range. The logic of Model 2 was structured on the recommendations of Cowled and Garner [38] who stated that a number of factors should be accounted for during disease modelling in wild pigs, including distribution and habitat connectivity, density, movements, social and group structure and age structure. Model 3 was a non-spatial herd model designed to replicate model 2, except that model 3 assumed no spatial relationships between wild pig herds. Comparison between model 2 and 3 allowed consideration of the importance of spatial relationships during simulated epidemics. See Additional file 1 for a method summary of model 3.

**Table 1:** Epidemiological parameters estimated for the between-herd model (parameters derived from the within herd model except arbitrary transmission probability)

Parameter	Lowest	Estimate	High	Distribution
Latent period	5	NA	9	Uniform
Infectious period	15	27	42	Triangular
Immune period	88	NA	475	Uniform
Probability of transmission between herds	NA	0.75	NA	NA
Proportion of herds where all members killed by CSF infection	NA	42%	NA	NA

### Study area, biology and distribution of wild pigs

**Figure 1:**
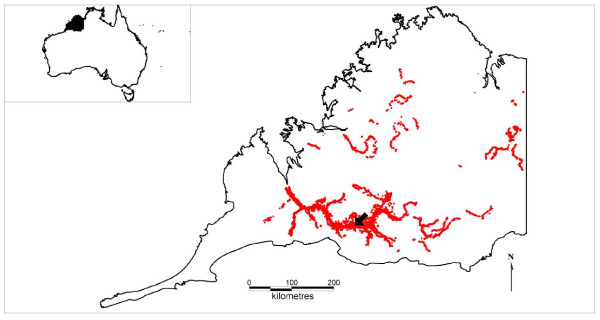
**Feral pig distribution in the Kimberley**. The red dots represent simulated pig herds within known wild pig distributions. The black arrow indicates the introduction site for all simulations. The inset displays the location of the Kimberley region in North West Australia.

The between-herd model was structured on a population of wild pigs in the Kimberley region of north-west Australia. The Kimberley region is a large (approximately 300 000 km^2^), remote and sparsely populated pastoral (cattle) region. Pigs were introduced by European settlers during the late 19^th ^century and subsequently became wild [39]. Questionnaire surveys were conducted across the Kimberley region to estimate wild pig distributions and densities and have been previously reported [40,41]. Wild pigs are currently found across approximately 26 000 km^2 ^of the Kimberley region. The population chosen for disease introduction was located in the Fitzroy River area (see study area and introduction site in Figure 1). Other researchers have investigated the biology of wild pigs in the region [42] and the population structure was typical of other wild pig populations, for example with groups comprised mostly of solitary boars or herds containing adult females and juveniles [42,43]. Average group sizes were generally small (mostly herds of 12 or less, but up to 30 pigs). In high density habitat, wild pigs may be present at approximately 3-8 pigs per km^2 ^(the range in density depending on whether an edge effect is taken into account) [42].

### Population at risk and habitat contiguity

Within the known wild pig distribution, permanent water sources (either linear water sources such as rivers or point sources such as dams) were identified and buffered (by 2 km) within a GIS (Mapinfo^® ^v. 10.5). These polygons represented the core habitat of wild pigs in the study area. Thus, a 16 701 km^2 ^area of "core" habitat was identified in the overall distribution of wild pigs (of 26 000 km^2^). This method was chosen to refine the distribution of wild pigs because the Kimberley region is a tropical ecosystem, is very hot (November mean maximum and minimum daily temperature are 41.0 and 25.6°C respectively [44]), and in accordance with field observations it is recognised that wild pigs in these conditions require at least daily access to water for survival [24]. Saunders and Kay [45] demonstrated daily home range lengths of approximately 2 km, and it was therefore assumed that wild pig home ranges must be located within 2 km of permanent water for survival (that is, a large wild pig home range must contain some of this high quality water habitat). Permanent water was identified using a spatial dataset from Geoscience Australia [46], with additional data layers of artificial water sources (stock water) supplied from the Department of Agriculture and Food, Western Australia (unpublished data).

The total number of wild pigs within each polygon was estimated by classifying the polygon as having a high, medium or low density of wild pigs [40]. Thus the 16 701 km2 area was divided into three polygons of 7563 km^2 ^(low density), 469 km^2 ^(medium density) and 8639 km^2 ^(high density). Relative densities were then quantified using published estimates. Choquenot et al. [24] reviewed wild pig densities in various habitats in northern Australia. These ranged from 1-20 pigs km^-2^. Twigg et al. [42] estimated 3 pigs km^-2 ^within a high density region of this study site. Given our study site was in a semi-arid region it was assumed that densities would be at the lower end of the range listed in Choquenot et al. [24]. Thus, estimates used for population densities were 1, 2 and 3 pigs per km^2 ^in low, medium and high density pig habitat, respectively. Our upper estimate is thus consistent with an edge effect modified estimate from prior work in our study area [42] and consistent with the lower estimate of Choquenot et al. [24].

The total population in each polygon was divided into groups. Group sizes and structure were estimated based on both published literature and unpublished data from the study area. Caley [47] found approximately 12% of a trapped population were males greater than 18 months of age. Thus 12% of the population was assumed to be solitary males. The remaining population was divided into social groups (female groups) with simulation using a *B *pert distribution of group sizes (minimum group size = 5, most likely = 7, maximum = 45) based on prior research on group sizes [24,42] and allowing for a slightly greater range due to the limited size of the study in Twigg et al. [42]. These groups were dispersed randomly in the core habitat (2 km buffered permanent water) whilst maintaining low, medium and high relative density classifications. This formed the final population data base for use in Model 2. The simulated distribution of wild pig herds (and solitary males) throughout the Kimberley region is shown in Figure 1.

### Classical swine fever

There are several reports detailing mortality and morbidity rates associated with CSF outbreaks in wild pigs. There appears to be a wide spectrum of clinical outcomes seen, with some outbreaks in wild pigs leading to very high mortality and morbidity, [34,48-50] with other caused by low or moderate virulence strains, especially in Europe [51-54].

South-east Asia has regions that are endemically infected with CSF but is also in relatively close proximity to our Australian study site. For geographical reasons south-east Asia may thus represent a potential source of an outbreak of CSF in wild pigs at our study site. The virulence of South-east Asian CSF strains are largely undocumented, but outbreaks in some islands with highly susceptible pig populations have lead to substantial mortality events and may therefore be due to moderate or highly virulent strains (Jenny-Ann Toribio, personal communication, July 2011).

Therefore, in this study, it was assumed that the virus was highly virulent, although a sensitivity scenario assuming a lower virulence was also conducted. In the highly virulent simulations, an individual case fatality rate of 90% associated with highly virulent CSF infection was assumed (within-herd model-see Additional file 1). This resulted in 42% of herds having all members die. Table 1 summarises the key epidemiological parameter estimates used in Model 2.

### Model 2: description of between-herd model

#### Model software

The model was reminiscent of a previous disease model in domestic animal populations [55-57]. Applications were coded in MapBasic^®^, and implemented in Mapinfo^® ^[58]. These software environments together represent a sophisticated and customisable geographical information system (GIS).

#### Classical swine fever transmission

The model's treatment of virus transmission can be considered in two ways, transition of individual herds between disease states temporally, and between herd transmission.

**Figure 2:**
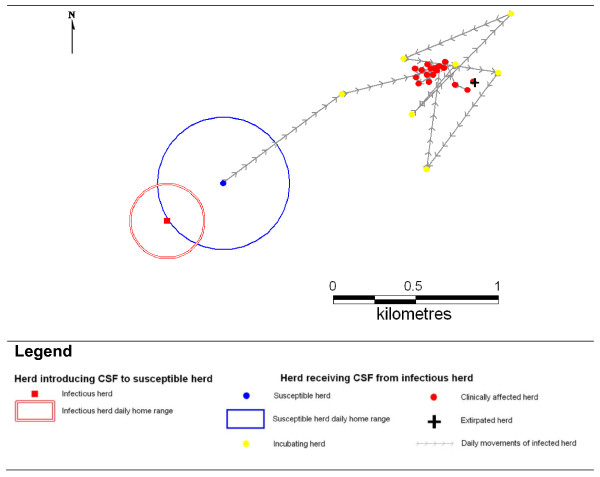
**Representation of a typical disease transmission event and subsequent daily movements of the newly infected herd in the process model**. Explanation: an infected herd (red square) and susceptible herd (blue circle) have overlapping daily home ranges (red and blue circles respectively). Classical Swine Fever transmission may occur according to an arbitrary probability. Following infection the incubating herd continues to move normally for several days (yellow dots) before becoming clinically affected (red dots) with shortened daily movements and eventually having all herd members killed (black cross). This infected herd does not contact another herd and CSF is not transmitted to another herd.

When a susceptible herd becomes exposed to virus it may become infected whereby it will progress through a latent, infectious and recovered sequence, although the herd may cease to exist if all members are killed by the infection (in the model this is simulated according to a probability derived from Model 1). The time spent in each state is stochastically determined using probability distributions. The time step used in the model is one day. To represent disease transmission between herds it is necessary to consider the spatial distribution of the wild pig population (see sections below). Local herds have the chance of coming into contact where their daily home ranges overlap, and if one of these herds is infected then there is a probability of virus transmission between an infected and un-infected herd. In the absence of good field information, this probability was arbitrarily set at 0.75, with a thorough sensitivity analysis undertaken to determine how the model outcomes change in response to changes in this parameter. Figure 2 shows diagrammatically how disease transmission between a single infected herd and neighbouring susceptible herd occurs. In this way, CSF can spread through a wild pig population comprising contiguous herds. However, CSF (particularly virulent strains) frequently produces severe clinical disease [59] that could be expected to affect the activity of feral pigs [60]. To allow for this, when wild pig herds are in the infected state, their mobility is assumed to decline to just 10% of their normal daily movement and home range (but sensitivity analyses occurred) (see Figure 2). This limits the probability of overlapping home ranges and thereby reduces the chance of transmitting virus to nearby uninfected herds.

#### Model logic (movements and home ranges)

Most ecological studies in Australia have demonstrated that wild pigs are relatively sedentary, within fixed home ranges, displaying little or no dispersal but moving small distances daily within their larger home range [24,61]. Females move smaller distances than males. However, a small but potentially epidemiologically important proportion of pigs may disperse longer distances [61-63].

To capture movements, each pig herd (including solitary males) was assigned an annual home range. This was simply a circle (buffer) around each location. There was considerable overlap in annual home ranges, reflecting the overlap that occurs in the field [45]. Pig herds were randomly moved a linear distance each day within each home range. Ninety-five percent of pig herds were constrained to movements within their own home range. It was assumed that since pig herds usually consist of females and offspring then published daily movements of females will represent the movements of groups, whilst the published daily movements of males will represent the movements of solitary pigs. Daily movement distances were estimated from the daily home ranges described by Caley [47]. After their daily movement, a daily home range of approximately 1 km^2 ^was structured around the herd's final location for the day [45,47]. See Table 2 for a description of ecological parameters used in Model 2.

**Table 2:** Ecological parameters estimated for the between-herd model

Parameter	Estimate	Highest	Lowest	Probability Distribution
Density (pigs km^-2^)	1-3	NA	NA	NA
Herd size^i^	7	45	5	*B *pert
Male home range (km^2^)^ii^	12	31.2	3.7	Triangular
Female home range (km^2^)^ii^	7	19.4	2.5	Triangular
Male daily home range^ii^	1.5	9.99	0.2	Triangular
Female daily home range (km^2^) ^ii^	0.9	3.6	0.06	Triangular
Male daily linear movements (km)^ii^	1	2	0.1	Triangular
Female daily linear movements (km)^ii^	0.7	1.8	0.1	Triangular

^i^12% of individuals were assumed to be solitary (mostly males), the rest of the population were distributed into female groups.

^ii^Caley [51].

A small percentage (5%) of pig herds were allowed to move a normal daily distance but were unrestrained by their home range and were able to move to adjacent core water habitat. In effect, this allowed a small proportion of pigs to disperse or display extraordinary movements. Giles [62] showed that groups of wild pigs can move 20-30 km during short periods in response to natural events such as flooding. Saunders and Bryant [64] showed that wild pigs can move from a study area in response to persecution, and that long range dispersal can occur. Caley [61] showed that a small proportion of wild pigs may move 20-30 km over several years (although the majority stay within their home ranges).

#### Model logic (surveillance)

A surveillance module was included to allow assessment of the surveillance of wild pigs for disease after an outbreak was discovered. The aim of surveillance was to delineate the infected area of the pig population following detection of the disease incursion [65]. Surveillance was simulated using realistic surveillance strategies and assumptions, and integrated with the control modules (see below).

A time to first detection of the outbreak was selected. At that point an index case was randomly chosen to be found, from all the infectious or recovered herds present at that time. A six week time to detection was arbitrarily chosen for simulations -- this is similar to other published estimates of potential time to first detection of FMD in Australian wild pigs [66] and detection of The Netherlands domestic outbreak [67]. Surveillance was assumed to begin three days after detection of the index case to allow organisation of surveillance resources. Surveillance was then structured around a user defined grid (a 10 × 10 km grid structured across the wild pig distribution in the Kimberley region was used). Surveillance was assumed to be conducted by aerial shooting from helicopters. Aerial shooting from helicopters is a well researched, effective and humane wild pig control and surveillance tool used in Australia [64]. The number of helicopters to be used (3), how many individual pigs that could be sampled by one helicopter team each day (70) and the area a helicopter can search each day (200 km^2^) were selected (based on author experience). Assuming 4-5 pigs are selected from each sampled herd (to give 95% confidence of detecting disease, where prevalence is assumed 50% [68]), the number of pig herds that can be sampled each day was determined. A sampling intensity was thus calculated, based on the number of herds that can be sampled each day and the average population of herds within a grid. Whether any given herd within a grid cell was actually sampled was probabilistically determined from the sampling intensity using Monte Carlo methods. A sampled herd was probabilistically categorised as infected based on defined test sensitivity (95%).

A circle two grid cells in radius surrounding an index cell (i.e. the index cell is the grid cell that contains the index case) was buffered and grid cells within this circle selected for surveillance. These cells were progressively sampled from closest to the index cell to furthest, each day depending on resources available. When all cells were sampled within the initial buffered region, and disease was present in at least one cell, the search area was expanded by another two grid cells and all grid cells were again sampled. This progressed until a final buffered area underwent surveillance and no infected cells were discovered. At this point an assumption was made that the epidemic was delineated.

#### Model logic (control)

Two control strategies were implemented in the model (aerial culling or vaccination) although only one of these methods could be selected during a single epidemic simulation. Control was assumed to begin after surveillance had finished delineating the infected area. For each control strategy, the infected area delineated during surveillance was buffered. The buffered area of land surrounding the infected area (herein control zone) was thus at least several pig home ranges wider than the "known" infected area. The control zone was constructed in this way to prevent migration of incubating or infected pig herds outside the infected area, and hence prevent spread of the epidemic to neighbouring susceptible populations.

To implement this, all grid cells within the control zone were ordered from the centre of the infected area outwards. If culling was the chosen control method, for each simulated day a portion of herds (priority from closest to the centre of the infected area to furthest) was culled. The proportion of the herds culled was defined by both the availability of control resources and the probability that a herd would be detected during aerial culling operations. The availability of control resources was measured by two parameters, the number of helicopters used for culling (4) and how many individual pigs could be culled each day (300) by each helicopter. The probability that a herd would be detected and culled during culling operations was user defined, with a default of 0.8 [64], meaning that 80% of herds would be randomly culled during baseline model simulations. The effect of culling a range of proportions of the population was assessed during experimentation and sensitivity analysis.

For the vaccination control option it was assumed an oral CSF vaccine that could be distributed aerially was available in Australia. In contrast to the culling option, pig herds were prioritised from furthest to closest to the zone centre. This approach was assumed to ensure that incubating pig herds had less opportunity to migrate infection beyond the immune buffer, before immunity developed. Similar to culling, there was a probability that each herd would be vaccinated, and the time to vaccinate herds in the control zone was determined by control resources available. A delay of 7-14 days until full herd vaccine immunity develops following vaccination was assumed [69,70]. Once immune, it was assumed that herds could not transmit virus.

During each simulation in which control was instigated, epidemics were classified as successfully or unsuccessfully contained and eradicated. In each control simulation the total infected area of a controlled epidemic was calculated and compared with the identical but uncontrolled scenario. If the final epidemic area during a controlled scenario was less than the same epidemic without control, and if eradication occurred, it was assumed that control measures had contained and eradicated an epidemic.

### Number of simulations

A vexed question for simulation modelling is how many simulations (or model runs) are required to produce a result of sufficient precision? Too many simulations are computationally inefficient. However, outputs from a stochastic simulation model have variability. If each simulation is considered one observation in a sample, it is important to have enough simulations (or a large enough sample size) to ensure that the estimate of the parameter of interest (${\hat{\theta }}_{n}$) approaches the true population value for the model (θ). If the sample size is large enough, the parameter estimate (${\hat{\theta }}_{n}$) generated from the model simulations converges with the true population value (θ) [71] for the model. Consistency (or convergence) can therefore be stated in relation to how the variance of the sample reduces to zero as the sample size approaches a theoretical infinity [71]:

$${\text{lim}}_{n\to \infty }V\left({\hat{\theta }}_{n}\right)=0.$$

Thus in practical terms, when a sample size increases such that the variance is minimised, ${\hat{\theta }}_{n}$ is close to the true θ. To estimate our sample size, we calculated the mean of the parameter-of-interest (after each simulation). We then determined the co-efficient of variation of this mean. At the point when the co-efficient of variation was less than 15% for 30 consecutive simulations we considered that convergence had occurred and that this number of simulations was adequate to estimate the parameter with precision. We repeated this process for every output parameter of the simulation model, and determined the maximum number of model simulations required across all output parameters. This number became our sample size (the number of simulations required).

### Sensitivity analyses and detection of interaction

Best estimates (as assumed following the literature review and detailed above) for all input parameters were assessed during baseline runs. For all baseline runs, sensitivity and experimental analyses, infection was introduced into the same wild pig herd (see Figure 1) to ensure a valid comparison of outputs. The major ecological, epidemiological and population parameters were varied systematically, by multiplying the best estimates one at a time by 0.25, 0.5, 0.75, 1 (best estimate) 1.5 and 2. An exception was made for transmission probability, in which the 1.5- and 2-times factors were excluded and 1.33-times (probability = 0.99) included to ensure the probability remained less than one. Parameters selected for sensitivity analyses were:

CSF transmission probability (between herds with overlapping home ranges)

Herd mortality rate (proportion of herds with all individuals dying of CSF)

Home range size

Daily linear movement distances

Proportion of population that can move extra-ordinary distances

Density (pigs km^-2^)

Reduction in movement of a clinically affected herd (proportion)

Outputs measured are listed in Table 3. All output measures underwent pair-wise linear regression against the area of the infected land to determine whether parameter outputs were correlated and whether a single output measure could be used for comparison during sensitivity analyses. Subsequently, the total area infected was used as the output parameter to represent the scale of the epidemic.

**Table 3:** Model outputs recorded during Classical Swine Fever simulations in wild pigs in north-west Australia

Output measure	Description	Best model prediction Median (95% probability intervals)
	**Outbreak description of highly virulent strain (without control or surveillance)**	

Proportion of introductions established (%)	The proportion of all simulations where a single point introduction leads to disease establishment (disease spreads to more than one herd)	0.98 (0.95-1.00)

Days to disease fade out	The number of days in which infected herds are present	759 (180-1424)

Infected herds	The total number of herds infected throughout the simulation	1302 (293-2707)

Total herds extirpated	The number of herds where every member died due to infection with CSF	563 (138-1146)

Incidence rate	The number of herds infected/day	1.86(1.14-2.73)

Area infected (km^2^)	The area of a minimum convex hull established around every infected herd throughout the epidemic	5979 (580-20537)

Area per day (km^2^/day)	The area of a minimum convex hull established around every infected herd throughout the epidemic/days of epidemic	9 (3-17)

Cumulative incidence	Proportion of herds infected (%) = The total number of infected herds/total herds in contiguous population	33 (14-70)

	**Low Virulence strain of CSF (without control or surveillance)**	

Proportion of introductions established (%)	As above	0.97 (0.94-1.00)
		
Days to disease fade out		976 (468-1442)
		
Infected herds		1829 (951-2825)
		
Total herds extirpated		184(96-288)
		
Incidence rate		1.84(1.24-2.63)
		
Area infected (km^2^)		11061 (2741-24393)
		
Area per day (km^2^/day)		11 (0-18)
		
Cumulative incidence		46 (23-84)

	**Non-spatial model**	

Proportion of introductions established (%)	As above	100%
		
Days to disease fade out		87 (86-90)
		
Infected herds		5304
		
Total herds extirpated		2234 (2205-2267)
		
Incidence rate		61 (58-62)
		
Area infected (km^2^)		NA
		
Area per day (km^2^/day)		NA
		
Cumulative incidence		100%

Best parameter estimates (most likely scenario), low virulence and non-spatial CSF scenarios shown.

Sensitivity analysis used an iterative process [72]: first input parameters were screened to identify influential and non-influential parameters, and then influential parameters for interaction were identified. Scatter plots and regression (linear and polynomial) were used to identify influential parameters. The most influential parameters identified during one at a time sensitivity analyses underwent factorial experiments and were tested for interactions by ANOVA. A full factorial experiment was conducted in which the total area of the epidemic (km^2^) was the response variable and explanatory variables identified in one at a time analyses were varied at three different levels (half, baseline and double parameter estimates). The factorial experiments occurred during simulations in which disease was assumed detected at 6 weeks post-introduction and control using aerial culling was instigated after delineation of infected areas (see culling scenarios for more information). ANOVA included testing for effects and for pair-wise and three way interactions.

### Scenarios analysed and model experimentation

Experimentation was conducted to examine the effects of disease ecology, surveillance and control options on the scale of an outbreak. Table 4 details the scenarios that were simulated.

**Table 4:** Model experimentation and scenarios analysed

Scenarios tested	Summary	Parameters varied from baseline
Baseline	Baseline parameters used. No surveillance or control used.	NA
Aerial culling	Baseline parameters used but culling introduced at variable intensities and culling zone widths.	Size of culling zone width: 10, 20, 30, 60, 100 km. Probability of culling a herd: 20, 40, 60, 80, 99%
Aerial vaccination	Baseline parameters used but vaccination introduced at variable intensities and vaccination zone widths.	Size of vaccination zone width: 10, 20, 30, 60, 100 km. Probability of vaccinating half a herd: 20, 40, 60, 80, 99**%**
Low virulence CSF	A CSF strain of moderate virulence was introduced.	The within herd model (Model 1) was used with 30% mortality assumption to generate new parameters for the between herd model.
		Herd immune period increased to 135, 666 4003 days (lowest, most likely, highest). Probability that all individuals in a herd die of CSF decreased (0.10).
Comparison between non-spatial and spatial modelling assumptions	A non-spatial model was parameterised as for the spatial modelling, except non-spatial disease transmission was assumed using a non-spatial, homogenously mixing population.	Disease transmission occurred homogenously using a probability derived from an equation rather than through spatial proximity (see Additional file 2). A baseline and culling scenario was conducted.

## Results

### Simulations required

**Figure 3:**
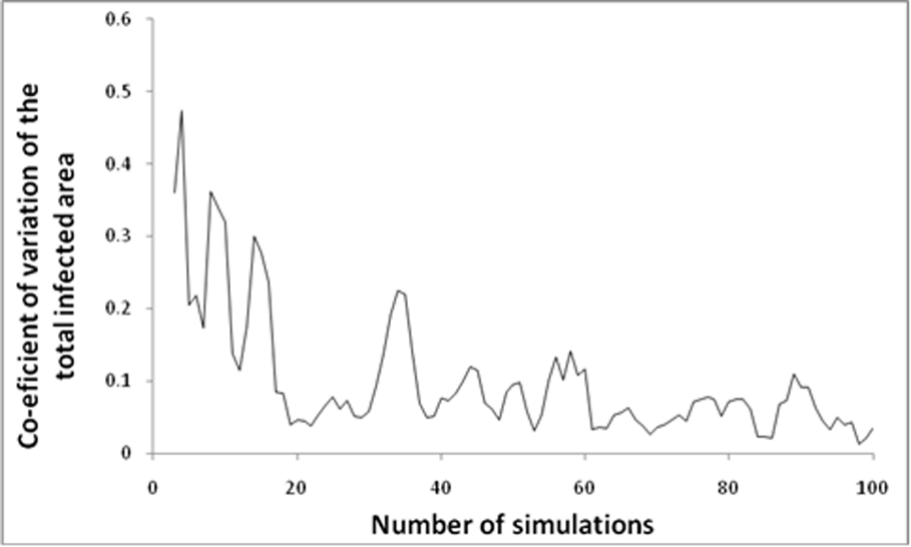
**Number of simulations plotted against coefficient of variation of the total infected area**. Approximate consistency (coefficient of variation < 15%) was achieved at 59 simulations.

Sample sizes required to achieve a low coefficient of variation for 30 consecutive simulations were calculated for all outputs. The maximum number of simulations required was 59 (for the output measure, total infected area). See Figure 3.

### Simulation results using best parameter estimates (and a highly virulent virus)

Using the best estimates for parameters, CSF generally established (98% of simulations) following a single point introduction. CSF generally progressed in an epidemic wave down contiguous habitat for several years, before dying out (when the epidemic arrived at the end of a contiguous sub-population or at a low density pig area). The daily herd incidence rate was low, despite epidemics that lasted several years, across thousands of square km and cumulatively infecting thousands of herds.

**Figure 4:**
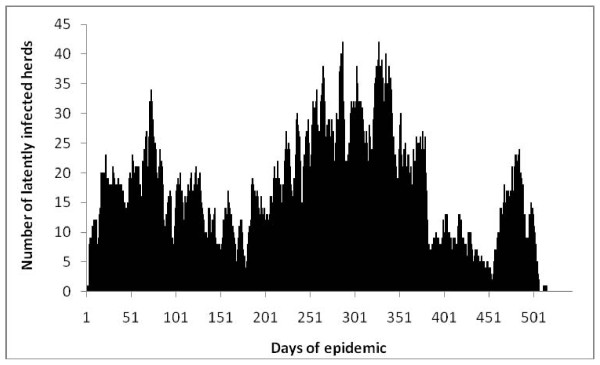
**A typical epidemic curve for one Classical Swine Fever simulation in wild pigs in north-west Australia**.

Table 3 lists and defines outputs and the results of the simulations. Figure 4 is a typical epidemic curve for one simulation. Additional file 2 is a PowerPoint presentation that shows a week by week progression of a typical simulated epidemic (Model 2).

### Sensitivity analyses

In general, output parameters were highly correlated with one another (*r *> 0.94), except for the number of immune animals and incidence rate (*r *< 0.1). Subsequently, the total area infected was arbitrarily chosen as the single output variable for comparison of parameter estimates during sensitivity analyses.

**Figure 5:**
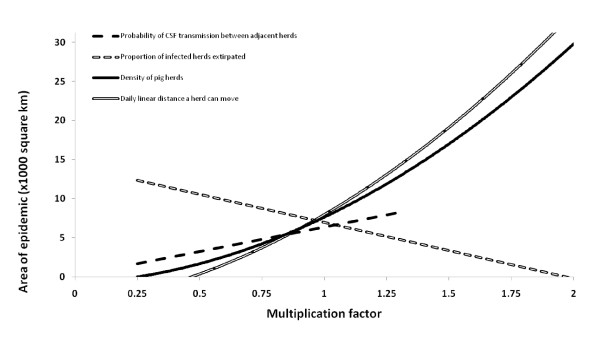
**Parameters with a large influence on Classical Swine Fever outbreaks in wild pigs identified during one at a time sensitivity analyses**. The figure shows the effect of changing each influential parameter on outbreak size. The correlation between outbreak size and the level of parameter was greater than 0.9 for all parameters (data points not shown). Multiplication factor of 1 was the best estimate/baseline.

Four parameters appeared influential (Figure 5 and Table 5). As the density of herds, the daily linear distance that a herd could move and probability of disease transmission between herds with overlapping daily home ranges increased, epidemics were larger. However, as the probability that all members of a herd would die from CSF infection (a proxy for CSF virulence) increased, epidemics became smaller. Parameters that had little predictable effect on epidemic size were the proportion of the herds that moved extraordinary distances, the yearly home range size of a pig herd and the reduction in the movements of a herd when it became clinically affected.

**Table 5:** The influence of changing parameters relative to baseline (multiplication factor, 1 = best estimate) on Classical Swine Fever outbreak size (000 km^2^) in wild pigs in north-west Australia

	Parameter						
	
Multiplication factor	Probability of Transmission	Mortality probability	Density	**Movers**^**2**^	Linear distance	Home range	Reduction in movement when sick
0.25	1.522	12.097	0	6.578	0.023	9.793	7.236
0.5	3.362	12.393	0.004	8.393	0.196	3.176	8.406
0.75	5.170	8.470	1.360	10.502	0.126	8.667	7.324
**1 (baseline)**	**5.979**	**5.979**	**5.979**	**5.979**	**5.979**	**5.979**	**5.979**
1.5	8.499^1^	5.960	20.46	10.242	25.180	16.832	9.645
2	NA	2.009	28.513	8.509	31.352	8.824	11.088

^1^Multiplication factor = 1.33 to ensure probability remained less than 1 (probability = 0.99).

^2^Proportion of herds able to disperse away from their starting home range.

### Interaction

Four influential factors were identified during one at a time sensitivity analyses. Since the density of herds and the daily linear distance a herd can move were highly correlated and changes in these variables produced near identical effects (see Figure 5), and to reduce the number of scenarios investigated during the factorial experiments the density of herds was not included during factorial experimentation. Thus, three influential variables (the daily linear distance a herd can move, probability of a herd dying due to CSF and probability of transmission of infection between overlapping herds) were varied at three levels resulting in 27 permutations for inclusion in analyses of the factorial experiment. The response variable (area of the epidemic) was not normally distributed and was log transformed, with the resulting transformed distribution being approximately normal (skewness = 0.298, kurtosis = 2.48).

In ANOVA all three main effects were significant and were significantly different from each other (*P *< 0.05) but no interactions were detected (*P *> 0.05).

### Scenarios and experimental results

#### Aerial culling

Based on the assumptions made, the surveillance program was able to consistently delineate the infected area within 1-2 weeks.

**Figure 6:**
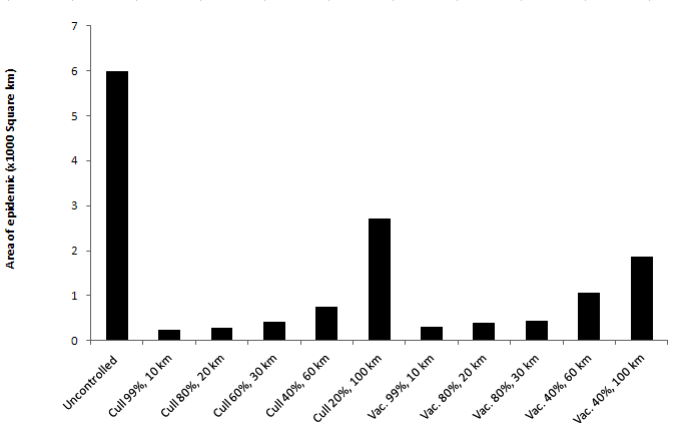
**A comparison between the median size of a classical swine fever epidemic in wild pigs in which no control, culling or vaccination was used**. Combinations displayed are the lowest proportion of culling or vaccination and lowest control zone widths in which successful containment and eradication was achieved in every simulation.

Culling was most successful at controlling the incursion when high proportions of herds were culled and control zones sizes were relatively large (see Table 6 and Figure 6), or when at least one variable was high (either a very wide culling zone or very high proportion of herds culled). Conversely, where culling proportions were low and control zone sizes small, containment and eradication attempts were less successful. If a realistic proportion of the herds could be culled - for example, > 60% of herds [64] - containment and eradication was achieved in all simulations in which the culling zone was sufficiently large (> 30 km width). Table 6 shows that there were critical combinations of culling proportion and control zone size above which disease could be contained and below which disease escaped in some simulations.

**Table 6:** Containment and eradication success following establishment of culling zones of varying intensity and size around surveillance delineated outbreaks of CSF in wild pigs

	Culling zone width (km)					
		10	20	30	60	100
Proportion of herds culled (%)	20	O	O	O	O	X
	40	O	O	O	X	X
	60	O	O	X	X	X
	80	O	X	X	X	X
	99	X	X	X	X	X

Scenarios in which combinations of culling level and culling zone width resulted in CSF containment and eradication of all outbreaks are marked with X, and scenarios where infection was not always contained are marked with O.

#### Vaccination

Similarly to culling, vaccination was most successful in scenarios in which a high proportion of herds were able to be vaccinated and vaccination zone widths were large (see Table 7 and Figure 6). Comparison of Table 6 with Table 7 shows similarities between the success of culling and vaccination. However, it was also evident that vaccination was less effective at containing and eradicating epidemics than culling, with a greater number of combinations of buffer sizes and widths resulting in some outbreaks unable to be contained. For example, assuming 99% cull at a buffer width of 10 km culling was successful but a 99% vaccination proportion with a 10 km vaccination zone was not. This is supported by Figure 6 in which the size of an epidemic was always smaller if culling was used rather than vaccination, even if containment and eradication was still achieved.

**Table 7:** Containment and eradication success following establishment of vaccination zones of varying intensity and size around surveillance delineated outbreaks of CSF in wild pigs

	Size of vaccination buffer (km)					
		10	20	30	60	100
Proportion of herds vaccinated (%)	20	O	O	O	O	O
	40	O	O	O	X	X
	60	O	O	O	X	X
	80	O	X	X	X	X
	99	O	X	X	X	X

Scenarios where combinations of vaccination level and vaccination zone width resulted in CSF containment and eradication are marked with X, and scenarios where infection was not contained are marked with O.

#### Low virulence strain

Outbreaks of a lower virulence CSF were longer in duration, spreading across a larger land area and infecting more herds (with a higher cumulative incidence). The lower virulence strain resulted in lower herd mortality but a similar incidence rate (see Table 4).

#### Non-spatial modelling

Under non-spatial modelling assumptions the epidemic progressed much more quickly than during spatial modelling (Table 3) - incidence rates of 61 versus approximately 2 herds/day, respectively. Outbreaks were of shorter duration (median 87 days versus several years, respectively), but with all herds being infected (100% versus 33% cumulative incidence, respectively). Control (assuming an 80% probability of herds being culled) was able to shorten the duration of epidemics (70 days until disease fadeout) but this required that a median of 3857 herds (73% of herds) be culled for eradication to occur (compared with 419 herds or 11% of herds required to be culled for disease eradication during spatial modelling).

## Discussion

This study highlights the critical importance of ecology, behaviour and spatial structuring of wildlife populations on the management of wildlife disease. It also provides some useful guidelines for containing and eradicating epidemics from widespread terrestrial vertebrate species such as the wild pig.

The spatial structuring evident in the wild pig population in our study area [40] had a large influence on simulated epidemics. For example, in our simulations, epidemics typically travelled in waves along the larger river systems along which wild pig meta-populations were located (see Additional file 2). This spatial population structuring resulted in a very low daily incidence rate (< 2 herds each day). In contrast, epidemics in non-spatial populations progressed much more quickly (~61 herds per day). This occurred because epidemics were not confined to that proportion of the population in the immediate vicinity of infected herds during non-spatial simulations, but instead could infect herds randomly throughout the entire population. That is, models that do not take realistic spatial structures into account may overestimate the rate at which a disease will spread and overestimate the size of an outbreak.

The spatial nature of epidemics is of critical importance when planning surveillance for wildlife disease. Despite typical epidemics infecting thousands of wild pig herds, across thousands of square kilometres and lasting several years, the spatial structure of the population meant the number of currently infected herds was just a handful each day and new infections were limited to those herds directly adjacent to the narrow epidemic front. This highlights potential difficulties in attempting to detect active infection in wild populations using agent identification techniques (such as culture or PCR) in which no carrier state exists and immunity to an infectious agent is long lived (e.g. FMD and CSF in wild pigs). This is in accordance with prior research [73]. In addition, given the rapid turnover of individuals in many wild populations (especially wild pigs [74]) the decline in herd immunity may be rapid, leaving a relatively narrow window to conduct even serological investigations. Taken together this suggests that surveillance based on detection and investigation of clinical events is likely to be an efficient approach to finding short lived infections. Alternatively, in situations in which passive detection and reporting cannot be relied upon (e.g. little or limited opportunity to observe the population) then a more structured surveillance approach would benefit from a very good understanding of risk to allow targeted and therefore efficient sampling.

These simulation results also suggest that the spatial structuring and behaviour of wild animal populations should have an influence on the design of containment and eradication programs. Wild pigs are relatively sedentary with a high fidelity to a home range [61]; only short daily movements are observed [45,47]. Populations in Australia are also predictably associated with riverine habitat [40,75]. Our findings indicate that epidemics would only spread relatively slowly across the landscape (~9 km^2 ^each day) and are containable with some relatively simple and well researched control methods such as aerial shooting [64]. An adequate design in our modelling was a control zone width of approximately 30 km around the infected area, in which 60% of herds could be culled (although other combinations of control zone width and proportions able to be culled were also adequate). Indeed, following simulated introductions of disease into the largest and highest density sub-population of wild pigs in the Kimberley region (Fitzroy River populations), disease could be eradicated by culling just a median of 419 herds per outbreak (representing approximately 11% of herds in the sub-population of interest - the Fitzroy River population).

In comparison, when culling was instigated in the non-spatial model, very large numbers of wild pig herds (median 3857 herds) were required to be culled for disease fadeout to occur. This was because culling was not targeted to the immediate vicinity of the epidemic but was randomly implemented across the wild pig population. This is likely the reason that our findings differ from those of earlier mathematical modelling studies in wild pigs indicating that very high culling levels (e.g. 95%) may be required over short time periods or that relatively high culling rates may be required for a number of years (e.g. 49% per annum) to eradicate a disease such as FMD in wild pigs [8]. However, our modelling results are in agreement with other simulation modelling studies informed by empirical field trials in other species. For example, in the Ethiopian wolf only small vaccination corridors were required to reduce disease transmission in spatially structured wildlife populations [76].

Disease control using vaccination was generally less effective than aerial culling in our simulations. Specifically, in every culling or vaccination combination (width of control zone and proportion of herds culled or vaccinated), culling lead to smaller epidemics than vaccination (see Figure 6) (although slight differences in program implementation should be noted: namely vaccination occurred from the outside in, culling from the inside out). Additionally, infection was routinely contained at lower combinations of control zone width and probability in the culling scenarios than the vaccination scenarios (Table 6 c.f. Table 7). The reasons for this could include the time taken for immunisation to become effective, resulting in pig herds continuing to become infected and continuing to transmit disease during vaccination programs compared to culling (in which a proportion of pig herds are immediately removed from the population). The effectiveness of culling provides countries like Australia, where wild pigs are a damaging, introduced invasive species and where control is mandated, a great advantage for controlling disease in wild pig populations. In other continents such as Europe where wild boar are a valued endemic species and effective aerial culling may not be as acceptable, there is greater reliance on less effective tools such as vaccination. This may be equally true during outbreaks in domestic pigs, in which ethical concerns force consideration of vaccination over culling [77].

Other authors have reviewed the factors contributing to persistence of CSF in wild boar populations [36]. They found that attenuation of CSF viruses to moderate virulence, as well as the size and structure of wild boar populations may affect persistence, and recommended further spatially explicit modelling. Our modelling supports their hypothesis, with epidemics induced under assumptions of lower virulence resulting in longer and larger epidemics with higher cumulative incidences. However, in both low virulence and high virulence scenarios, without further introductions infection always died out after several years because as an epidemic front reached the end of a contiguous wild pig population there were insufficient susceptible hosts to maintain the infection. Infection could not spread back along the previous route of the epidemic because these herds had either died from CSF or were immune. These results concur with the majority of published studies investigating the nature of CSF outbreaks in European wild boar populations, in which CSF outbreaks generally fade out after several years [32,50].

This research may assist in the elucidation or confirmation of some important factors that influence the size, scale and behaviour of epidemics. Our results suggest density, the daily linear distance a herd can move, the probability of herd death from CSF and the probability of transmission between herds can have a large influence on the size and scale of an epidemic. Addressing each of these in turn, it appears that density and daily movements are highly correlated. This indicates that at higher densities, or with greater daily movements, larger epidemics occur because infectious herds are more likely to come into contact with other susceptible herds. Arbitrarily increasing the probability of transmission also increased the size of epidemics for similar reasons. In contrast to this, as the probability that a herd will die following infection increased, epidemics declined in size. This was likely because herds were eliminated before they had a chance to transmit infection to nearby herds. However, having a small proportion of the population that are unrestrained by assumptions of home range fidelity or varying the size of a herd's annual home range made little difference to the overall scale of the epidemic. Several authors have found that Australian wild pigs have a high home range fidelity [61,62,78], but have also found that a small proportion of pigs can move much greater distances and are unrestrained to home ranges [61,62]. Based on these modelling results it appears that these individuals would have little influence on disease spread, potentially because the probability that one of these individuals is incubating disease is low, and because clinical impacts of CSF reduce long range, aberrant movements anyway.

As with any simulation modelling, our results are very much dependant on the assumptions made. One of our main assumptions was that there are no artificial, human derived movements of infected wild pigs or contaminated fomites in the study area. Given that the Kimberley region is one of the least densely human populated regions in the world, and that there is no commercial pig production, we believe this assumption to be valid. However, were human mediated movements found to be important for transmitting infection, it is likely that control and surveillance programs would be made considerably more difficult. Although there is no field or published evidence of wild pigs chronically infected with CSF [37], Kramer-Schadt et al. [37] supposed that this may have been missed and assumed chronically infected pigs for the purposes of modelling. They used modelling to demonstrate that the existence of chronically infected wild boar may be a plausible mechanism to explain persistence of infection in a region. Other evidence [79] suggests that persistently infected piglets are critical in sustaining infection. Our modelling assumed chronically infected wild pigs, but only allowed for the longest recorded wild boar piglet infectious periods of 39 days, [80] which is shorter than has been recorded in domestic pigs [50].

In conclusion, our modelling has captured many of the important factors that are likely to influence epidemic behaviour in wild pig populations. Our results indicate that spatial structuring of wild pig populations is an extremely important feature. Density, daily movement distances, disease-induced herd mortality rates and transmission probabilities between adjacent herds are also important. It is also evident that control and surveillance programs should account for the spatial structuring of wild populations, and that it may only be necessary to cull or vaccinate relatively small proportions of a population to successfully contain and eradicate wildlife disease epidemics.

## Competing interests

The authors declare that they have no competing interests.

## Authors' contributions

BC, GG and KN designed the model logic. BC and GG coded the model. BC, KN and MW conducted analyses. BC prepared the first draft of the manuscript with contributions from KN, MW and GG. MW and GG edited the manuscript. All authors read and approved the final manuscript.

## Supplementary Material

Additional file 1 **A description of model 1 (within herd model) and model 2 (non-spatial model)**. This section provides a description of the model logic, parameters, references and coding steps used to parameterise models 1 and 3 [81-96]. Click here for file

Additional file 2 **A time series of a typical simulated epidemic**. This is a PowerPoint presentation of a typical simulated epidemic produced by model 2 (the spatial model). Click here for file
