# Supplementary material for: Controlling disease outbreaks in wildlife using limited culling: modelling classical swine fever incursions in wild pigs in Australia
Source: Vet Res. 2012 Jan 16;43(1):3. doi: 10.1186/1297-9716-43-3 (PMC3311561; doi:10.1186/1297-9716-43-3)
Supplement: Additional file 1 — A description of model 1 (within herd model) and model 2 (non-spatial model). This section provides a description of the model logic, parameters, references and coding steps used to parameterise models 1 and 3 [81-96]. [file 1297-9716-43-3-S1.DOC]

**Additional files 2**

*Model 1: description of within-herd model logic*

Wild pig populations predominantly consist of individual pigs (usually solitary males) or small groups [24, 43] and an epidemiological model should reflect this population structure [38]. An important epidemiological consideration is how group structure will influence key epidemic parameters (for example the infectious or immune period of a herd may be different to those of an individual pig). The purpose of this model was to determine the length of time a pig herd would remain infectious, the period of time before the herd would again become susceptible to infection and the proportion of herds where all members of the herd are killed by infection. These parameter estimates then informed the larger and more complex inter-herd model.

The within-herd model was a non-spatial, stochastic, state transition simulation model. It used mathematical and logical rules to numerically describe epidemics in single groups of wild pigs and assumed homogenous mixing. Homogenously mixing individuals within herds were assumed for several reasons: Wild pigs are gregarious, but not to the point of forming large mobs [43]; the majority of wild pig groups are small in tropical north Australia, the study site for this model [42, 63, 81], ensuring wild pigs contact other members of their herd each day; wild pigs are highly social, engaging in frequent social activity [43] resulting in groups of closely related individuals [62].

The model simulates disease transmission in discrete daily time steps. The model first simulated the size of a wild pig herd. Infection was then introduced to a single susceptible pig. Individuals transitioned within a susceptible-latent-infectious-recovered (SLIR) framework. Infection was transmitted between wild pigs according to equation 1 after Miller [82]. Wild pigs then transitioned through the SLIR framework according to simulated time periods associated with latent, infectious and immune periods. The model also included several biologically important features such as passively acquired immunity and infection related mortality. Model parameters are listed in Table 1. The rate of spread will depend on the effective contact rate, which is defined as the number of individuals coming in contact with an infectious individual per day sufficiently closely that transmission of infection could occur (James and Rossiter 1989). Key parameter estimates used were effective contact rate (20 feral pigs per day), herd immunity at which infection can re-establish (50%) and probability of mortality after infection (80%) (see Table 1 for further discussion of these parameters). One thousand simulations were arbitrarily chosen for the within-herd model.

| 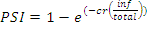 | Equation 1 |
| --- | --- |

Where PSI = probability a susceptible pig will become infected

cr = effective contact rate (20)

inf = number of infectious pigs in the herd

total = total pigs in herd

Model verification ensures that the programming and implementation of the conceptual model occurs correctly [83, 84]. Model verification occurred in several ways. Authors (BC and GG) carefully coded the models and checked each others work. Inbuilt error trapping features in MapBasic were used to check for compilation and run time errors. Finally, runtime and output parameters were checked systematically during simulation to verify that the model was performing as expected. When unusual results were detected, model code was checked.

*Model 3: Description of between herd non-spatial model*

Model 3 was a stochastic state-transition simulation model coded similarly to the spatial model (Model 2). Epidemiological parameters (e.g. herd infectious periods etc) and the population size (5304 herds) were exactly the same as for the spatial model (model 2) to allow comparisons between the non-spatial and spatial models.

The only difference between the spatial (model 2) and this non-spatial herd model (model 3) was its treatment of disease transmission. In model 2 (the spatial model), herds are placed spatially across the landscape and spatial proximity and movements according to assumptions of ecology and behaviour and a probability of transmission between herds with overlapping daily home ranges drives epidemic behaviour. In the non-spatial model, disease transmission to each herd was modelled using the equation of Miller[82] and James and Rossiter[85] and hence was completely non-spatial. The contact rate within the equation was estimated at 2. This contact rate was estimated from R_0_ values from the spatial simulations, taking an average over the first seven days of spread after Garner et al. [86]. The use of the same epidemiological parameters, estimated R_0_ from the spatial model and an non-spatial equation for disease transmission ensured that the only feature which varied between model 2 and 3 was the influence of space (and hence implicitly behaviour and ecology) on disease transmission.

Simulations of the non-spatial model (model 3) included baseline runs where no control was instigated and the model was run until disease ‘burnt out’ i.e. no infectious or latent groups were left in the population. Again, 59 simulations were conducted. A second series of simulations were conducted and included culling. In this scenario, it was again assumed that the epidemic was detected at 6 weeks and culling using the same parameters for the number of herds that could be culled each day (see culling scenarios in model 2) was conducted. A probability of culling of 80% was selected. By comparison between aspatial and spatial culling (i.e. model 3 and 2 respectively) exploration of the importance of the relative influence of spatial structure on control programs could be investigated.

Table 1: Parameter estimates used for a within-herd simulation model of Classical Swine Fever disease transmission within wild pig herds in north western Australia. Outputs were incorporated into a between herd model.

| **Epidemiological or ecological feature** | **Parameter estimates** | **Method of incorporation** |
| --- | --- | --- |
| Herd size | Most common group size in wild pigs in the study site: 1-2 sows per group with 5-12 piglets each, maximum 30 individuals [42]. Adult males solitary. These findings are similar to other published research [42]. | Herd size was simulated using Monte Carlo methods and a β-Pert distribution. Parameters: minimum=2 pigs, most likely=10 pigs, maximum=45 pigs. |
| Birth rate | Approximately 17% of population in groups are adult females [42]. Two to 0.85 litters per year are possible, but in seasonal breeding areas, approximately 1 litter per year is common[24, 87]. The study area (Kimberley region) has a monsoon and seasonality is evident in breeding pigs [42]. An assumption of 1 litter per year was made. Therefore probability that an individual in a group gives birth is 0.17 x 1/365 = 0.0004 | Group represented by an array in the model. Array size increases according to births (decreases with deaths). Herd immunity subsequently changes. The birth rate was imposed uniformly due to reduce modelling complexity. |
| Effective contact rate | It is assumed that each pig has close physical contact with 20 other pigs each day. | Incorporated in equation 1. |
| Latent period | 5-9 days (period from infection to first detection of virus in blood or other secretions by standard virus isolation techniques), based on the modal reported range for infection in wild pigs [88-91]* | Uniform distribution (5-9) used to select an incubation period for each pig in the array. Incubation period reduced each day of the epidemic |
| Infectious period | Most likely 15 days, but ranging from 7-25 days in postnatal infection in wild pigs [88, 91]. However, prenatal infection may lead to persistently infected piglets, shedding virus for extended periods: the maximum survival and viraemic period published for a persistently infected wild piglet is recorded at 39 days[88]. These individuals are epidemiologically important in helping sustain infection in wild boar and so were incorporated in the infectious period parameter using a beta pert distribution to reflect that they may only be a small proportion of infected animals [79]*. | Simulation using Monte Carlo methods and a β-Pert distribution (parameters: minimum=7, most likely= 15, maximum=39 days) used to select an infectious period for each pig in the array. Infectious period reduced each day of the epidemic |
| Immune period | 6 months to life Matthaeus and Korn [92] quoted in Artois *et al. [50]* and the maximal lifespan reported for wild pigs is 11 years [93]. | Uniform distribution (182- 4015) used to select an immune period for each pig in the array. Immune period reduced each day of the simulation. |
| Risk of mortality | High mortality rates, around 90-100% have been found in experimental infections with highly virulent CSF strains in wild pigs [88, 89, 91] and analysis of empirical data from a Pakistan epidemic [34] indicated that a mortality risk of 17-81% may have occurred and US channel island epidemics had very high mortality with 80% of the population dying [49]. Artois *et al.[50]* also quotes ‘mortality rates’ as high as 90% in wild boar in Europe.  Mortality rates above were interpreted as the risk of mortality*. | A triangular distribution case fatality rate was simulated with most likely 90%, lowest 85% and highest 95%.  For the low virulence scenario, repeated analyses were conducted. |
| Passive immunity | 6-16 weeks, since Vandeputte *et al.* [94] demonstrated that colostral immunity had waned by 7 weeks, allowing effective vaccination, although other authors found immunity extended to 16 weeks [37, 95]. | Each herd simulation continues until 50% of the herd are again susceptible. Piglets are added to the susceptible pool within the herd a number of weeks (triangular distribution, 6, 12, 16) weeks after birth (since all are born from previously infected sows). |
| Herd immunity | Kaden *et al.* [96] found that virus became undetectable in wild boar populations when herd immunity reached 49-60.3% and it has been suggested that the threshold for herd immunity may vary from 9-53%, or from 35-54% [32]. It was therefore estimated for this model that once herd immunity waned to less than 50%, herds were once again susceptible to infection. | Each iteration of the within-herd model continues until each herd has been through its epidemic and its immunity wanes to less than 50%. Herd immunity calculated as seroconverted pigs/total pigs, where total pigs is the sum of live pigs after the epidemic plus the newly born pigs minus pigs that have died of old age. |

*These parameters were based on an assumed infection with a highly virulent CSF strain, causing acute, fatal disease.
