# Supplementary material for: Controlling disease outbreaks in wildlife using limited culling: modelling classical swine fever incursions in wild pigs in Australia
Source: Vet Res. 2012 Jan 16;43(1):3. doi: 10.1186/1297-9716-43-3 (PMC3311561; doi:10.1186/1297-9716-43-3)
Supplement: Additional file 2 — A time series of a typical simulated epidemic. This is a PowerPoint presentation of a typical simulated epidemic produced by model 2 (the spatial model). [file 1297-9716-43-3-S2.DOC]

**Additional files 2**
